# Genetic evidence challenges the native status of a threatened freshwater fish (*Carassius carassius*) in England

**DOI:** 10.1002/ece3.2831

**Published:** 2017-03-22

**Authors:** Daniel L. Jeffries, Gordon H. Copp, Gregory E. Maes, Lori Lawson Handley, Carl D. Sayer, Bernd Hänfling

**Affiliations:** ^1^ Evolutionary Biology GroupSchool of Biological, Biomedical and Environmental Sciences, Hardy BuildingUniversity of HullHullUK; ^2^ Salmon and Freshwater TeamCefasLowestoftSuffolkUK; ^3^ Department of Ecology and EvolutionUniversity of LausanneLausanneSwitzerland; ^4^ Department of Life and Environmental SciencesFaculty of Science and TechnologyBournemouth UniversityPooleUK; ^5^ Laboratory of Biodiversity and Evolutionary GenomicsUniversity of LeuvenLeuvenBelgium; ^6^ Laboratory for Cytogenetics and Genome ResearchCentre for Human GeneticsGenomics CoreUniversity of Leuven(KU Leuven), 3000 LeuvenBelgium; ^7^ Pond Restoratation Research GroupDepartment of GeographyEnvironmental Change Research CentreUniversity College LondonLondonUK

**Keywords:** Approximate Bayesian Computation, introduced species, land bridge, microsatellites, postglacial recolonization

## Abstract

A fundamental consideration for the conservation of a species is the extent of its native range, that is, regions naturally colonized. However, both natural processes and human‐mediated introductions can drive species distribution shifts. Ruling out the human‐mediated introduction of a species into a given region is vital for its conservation, but remains a significant challenge in most cases. The crucian carp *Carassius carassius* (L.) is a threatened freshwater fish thought to be native to much of Europe. However, its native status in England is based only on anecdotal evidence. Here, we devise an approach that can be used to empirically test the native status of English fauna. We use this approach, along with 13 microsatellite loci, population structure analyses, and Approximate Bayesian Computation (ABC), to test hypotheses for the origins of *C. carassius* in England. Contrary to the current consensus, we find strong support for the human‐mediated introduction of *C. carassius* into England during the 15th century. This result stimulates an interesting and timely debate surrounding motivations for the conservation of species. We discuss this topic, and the potential for continued conservation of *C. carassius* in England, despite its non‐native origins.

## 1 Introduction

Obtaining a detailed understanding of a species’ native range and the distribution of its diversity within that range is fundamental for species conservation (Frankham, Briscoe, & Ballou, 2002; IUCN 2016; Reed & Frankham, 2003; Scoble & Lowe, 2010). A species is usually considered native if it has colonized an area naturally, whereas it is considered non‐native in areas which have been colonized with human intervention (Copp et al., 2005; Gozlan, Britton, Cowx, & Copp, 2010).

Species’ ranges are not static but often change dramatically over time in response to changing environments, newly‐arising dispersal corridors and human‐mediated factors. During the last 2.5 MY, the ranges of European biota have been most strongly impacted by glacial cycles (Hewitt, 1999). These processes have been extensively studied, particularly in freshwater fishes, whose postglacial recolonization dynamics have been determined by the history of river drainage systems (Bănărescu, 1990, 1992; Bernatchez & Wilson, 1998; Bianco, 1990; Hänfling & Brandl, 1998; Jeffries et al., 2016; Reyjol et al., 2006). For example, ephemeral rivers and periglacial lakes that result from glacial meltwater have provided opportunities for fish colonizations (Gibbard, Rose, & Bridgland, 1988) of otherwise isolated drainages (Arkhipov, Ehlers, Johnson, & Wright, 1995; Grosswald, 1980). However, the current distributions of European freshwater fishes have also been significantly impacted by human‐mediated translocations, which have enabled some species to overcome natural dispersal barriers like watersheds (Copp et al., 2005; Gozlan et al., 2010). Knowing whether an organism's contemporary distribution is the result of natural or human‐mediated dispersal can therefore have profound implications for its management (e.g., Copp et al., 2005).

The distinction between natural or human‐assisted colonization scenarios is often difficult to make, and this is certainly the case for much of the fauna of the British Isles. With very few exceptions (e.g., groundwater invertebrates McInerney et al., 2014; cold adapted fish species Hänfling, Hellemans, Volckaert, & Carvalho, 2002), it is thought that the vast majority of the species currently considered native in the UK have recolonized this region over the last 18,000 years, after the Weichselian ice sheet began to recede. In the case of primary freshwater fishes, this was made possible by connections between English and Continental river systems in Doggerland, the land bridge which existed between southeast England and Continental Europe. However, this window of re‐colonization opportunity was relatively short, as Doggerland was inundated at around 7,800 YBP when sea levels rose due to the melting of the Weichselian ice sheet (Coles, 2000).

After the loss of the Doggerland land bridge, the only means by which freshwater fish species (and indeed many other species limited to land or freshwater) could colonize the UK, precluding the very unlikely possibility of fertilized eggs being transported by migrating waterfowl (for which no empirical evidence exists, to our knowledge), would have been via human‐mediated introductions. The earliest known record of live fish translocations into the UK was the movement of common carp, *Cyprinus carpio*, into the southeast of England by monks in the 15th century (Lever, 1977). However, it cannot be ruled out that they were introduced by earlier civilizations, for example, the Romans, in the 1st century A.D or in the following few centuries by Viking invaders. These dates allow us to make a clear distinction between the possible arrival times, of a primary freshwater fish in the UK under two hypotheses; if native, then natural colonization must have occurred prior to 7,800 YBP, if introduced, then realistically it could not have arrived earlier than the arrival of the Romans, *circa* 2,000 YBP.

One species with a contentious status in the UK is the crucian carp *Carassius carassius* (Linneaus 1758), a freshwater fish native to much of central and eastern Europe. Currently, *C. carassius* is thought to be native in to southeast England, a consensus which is largely based on its distribution in southeast England (Marlborough, 1965; Marlborough, 1966), which closely matches those of other freshwater fish species thought to be native, such as silver bream *Blicca bjoerka* (L.), ruffe *Gymnocephalus cernuus* (L.), burbot *Lota lota* (L.), and spined loach *Corbitis taenia* (L.) (Wheeler, 1977, 2000). Archeological evidence, that is, *C. carassius* pharyngeal bones found at a single Roman archeological dig site in Southwark, London (Jones, 1978; Lever, 1977), also suggests that the species has been present in the UK almost 2,000 YBP. In contrast, however, Maitland (1972, 2000, 2004) suggested that *C. carassius* was introduced to south east England along with *C. carpio* in the 15th century, due to its absence in literature (e.g., Walton 1653 re‐published in 1987) until after the introduction of *C. carpio* (e.g., Houghton, 1895; Pennant, 1766; see also Rolfe, 2010). More recently, Jeffries et al. (2016) inferred substantial shared ancestry between UK and several Belgian and German populations from microsatellite and genomewide SNP markers, supporting the hypothesis of a more recent origin.

The correct designation of *C. carassius* as native or introduced in England is particularly important in light of sharp declines in the number and sizes of populations throughout Europe in recent times (Copp, Tarkan, Godard, Edmonds, & Wesley, 2010; Mezhzherin, Kokodii, Kulish, Verlatii, & Fedorenko, 2012; Rylková, Kalous, Bohlen, Lamatsch, & Petrtýl, 2013; Savini et al., 2010; Sayer et al., 2011). The threats to *C. carassius* are becoming increasingly recognized, as is shown by the inclusion of the species in a number of national red lists, for example, Czech Republic (Lusk, Hanel, & Luskova, 2004), Austria (Wolfram & Mikschi, 2007), Croatia (Mrakovčić, Buj, & Mustafić, 2007), and Serbia (Simic, Simic, Cirkovic, & Pantovic, 2009). But despite this, there are still very few active conservation initiatives for *C. carassius* in Europe. One of the most comprehensive of these exists in Norfolk, England, where the species has been designated as a Biodiversity Action Priority (Copp & Sayer, 2010; Sayer et al., 2011). However, given the conflicting views and lack of concrete evidence to underpin the native status of *C. carassius* in England, the question remains; is *C. carassius* native to the UK, or is its presence the result of human‐mediated translocations?

Such phylogeographic questions are difficult to test. Past approaches have included the use of simple molecular clock calibrations, whereby the amount of molecular diversity that has arisen between two lineages is known (from either fossil records or from vicariance) to have occurred within a certain amount of time (e.g., Voelker, 1999; Weir & Schluter, 2008). Such methods have proved extremely valuable in validating biogeographical hypotheses (Betancur & Armbruster, 2009; Fromhage, Vences, & Veith, 2004); however, owing to low eukaryotic mutation rates, long generation times and a lack of calibration points, these approaches cannot be employed for hypothesis testing over timescales of tens of thousands of years (Hedges & Kumar, 2003), as in this study. Recently, these challenges have been overcome by advances in molecular data analysis such as Approximate Bayesian Computation (ABC, Cornuet et al., 2008), which allows such questions to be addressed in a population genetic framework suitable for investigating events on a post‐Pleistocene timescale (e.g., Pedreschi et al., 2014).

In this study, we devise an approach that can be used to empirically test the native status of fauna in England (and indeed, the rest of the UK). This method capitalizes on the time constraints imposed on natural colonization of the UK by the existence or absence of the Doggerland land bridge. We use this method, along with ABC and highly polymorphic microsatellite markers, to test the status of *C. carassius* in England. Specifically, we test three possible alternative hypotheses for the *C. carassius* colonization: (1) all English populations originate from natural colonizations from Continental Europe more than 7,800 YBP; (2) all English populations were introduced by humans from Continental Europe sometime in the last 2,000 years; or (3) some English populations are native and some have been more recently introduced. Our ultimate aim is to increase the knowledge available for the assessment of status and conservation of *C. carassius* in England and Continental Europe.

## 2 Methods

### 2.1 Samples and molecular methods

**Figure 1:**
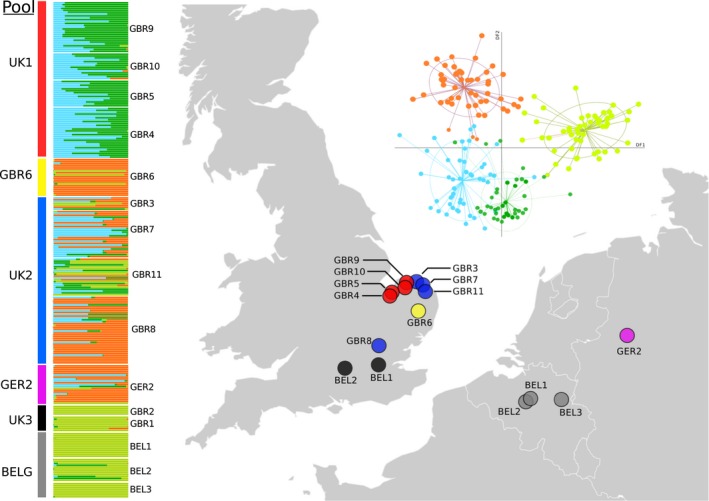
Discriminant Analyses of Principal Components (DAPC) analysis of *Carassius carassius* in northwest Europe showing similar genetic composition of English and Continental populations. Individual cluster assignments are shown in the left panel with the pool to which they are assigned denoted by the colored bars to the far left. Pool colors correspond to map locations and to the DIYABC scenario schematic in Figure 3

The samples used in this study include 257 *C. carassius*, from 11 populations from southeast England, three Belgian populations and one German population (Table 1, Figure 1). These represent a subset of samples from a Europe‐wide phylogeographic study, which used the same 13 microsatellite loci as used here, as well as mitochondrial DNA sequences and genomewide SNP data (see Jeffries et al., 2016 for Methods). All 257 samples were robustly identified as pure *C. carassius* with no signs of hybridization, with two abundant non‐native species *C. carpio* (L.) and *Carassius auratus* (L.) (Jeffries et al., 2016). Population structure analyses of the Europe‐wide dataset in Jeffries et al. (2016) showed that the four Continental populations in the current study are the most closely related (out of 49 populations from 12 countries) to those in England. These Continental samples fall at the end of an isolation by distance gradient that exists in Europe, which resulted from their east‐to‐west recolonization of Europe from glacial refugia (Jeffries et al., 2016). It is therefore highly likely that these populations contain the genetic variation native to these regions and are not the result of long distance human introductions themselves. Thus, if English populations did naturally colonize across the Doggerland land bridge, then the Belgian and German samples used in the present study are the most likely of all sampled populations in Jeffries et al. (2016) to represent the source of the British colonization.

**Table 1:** Location, number, and population diversity statistics of samples used in this study for microsatellite analyses

Code^a^	Location	Country	DIYABC Pool	Drainage	Coordinates		*N*	*H*_obs_	*H*_exp_	*A*_r_
					Lat	Long				
GBR1	London	UK	UK3	River Thames	51.5	0.13	9	0.11	0.08	1.33
GBR2	Reading	UK	UK3	River Thames	51.45	−0.97	4	0.03	0.03	NA
GBR3	Norfolk	UK	UK2	UK	52.86	1.16	7	0.16	0.08	1.48
GBR4	Norfolk	UK	UK1	UK	52.77	0.75	27	0.12	0.13	1.26
GBR5	Norfolk	UK	UK1	UK	52.77	0.76	14	0.13	0.18	1.3
GBR6	Norfolk	UK	RM	UK	52.54	0.93	20	0.22	0.17	1.55
GBR7	Norfolk	UK	UK1	UK	52.9	1.15	24	0.15	0.38	1.44
GBR8	Hertfordshire	UK	UK2	River Thames	52.89	1.1	37	0.16	0.15	1.43
GBR9	Norfolk	UK	UK1	UK	52.8	1.1	27	0.09	0.17	1.27
GBR10	Norfolk	UK	UK1	UK	52.89	1.1	14	0.21	0.16	1.69
GBR11	Norfolk	UK	UK2	UK	52.92	1.16	20	0.18	0.09	1.55
BEL1	Bokrijk	Belgium	BELG	River Scheldt	50.95	5.41	13	0.15	0.20	1.42
BEL2	Meer van Weerde1	Belgium	BELG	River Scheldt	50.97	4.48	12	0.19	0.11	1.48
BEL3	Meer van Weerde2	Belgium	BELG	River Scheldt	50.97	4.48	8	0.16	0.20	1.47
GER2	Münster	Germany	FFG	River Rhine	51.89	7.56	21	0.4	0.19	2.37
							257			

a Codes correspond to those in Jeffries et al. (2016).

DNA was extracted from fin clips and samples were genotyped at 13 microsatellite loci using the procedures described in Jeffries et al. (2016).

### 2.2 Standard population statistics

First, the data were tested for allele dropout and the presence of null alleles using Microchecker (Van Oosterhout, Hutchinson, Wills, & Shipley, 2004). FSTAT v. 2.9.3.2 (Goudet, 2001) was then used to check for linkage disequilibrium (LD) between loci, and to calculate *F*_IS_ and deviations from Hardy‐Weinberg equilibrium (HWE) for all loci and populations. Genetic diversity within loci and populations was estimated using Nei's estimator of gene diversity (*H*_e_) (Nei, 1987) and allelic richness (*A*_r_), which was standardized to the smallest sample size (*n* = 7) using the rarefaction method (Petit, El Mousadik, & Pons, 1998). In order to quantify differentiation among populations, pairwise *F*_ST_ values were calculated, also in FSTAT, using the multilocus *F*_ST_ estimator (Weir & Cockerham, 1984). Sequential Bonferroni correction and permutation tests (2,100 permutations) were used to calculate *p*‐values for *F*_ST_, and 1,000 bootstraps were used to calculate 95% confidence intervals. In addition, the significance of population divergence was also assessed through tests of allele frequency homogeneity among populations. This was performed in Genepop (Raymond & Rousset, 1995; Rousset, 2008) using the *G* test, which computes the *p‐*value for the observed differences in frequencies between populations for all loci, using a Markov chain algorithm. The R (R Core Team 2013) package, Adegenet v1.6 (Jombart & Ahmed, 2011), was used to test for isolation by distance using a Mantel test. Lastly, we used the Hierfstat package (Goudet, 2005) in R to quantify genetic variation (*F*_ST_) at four hierarchical levels of population isolation, the population‐level (separate ponds within countries), the country‐level (between Belgium and Germany), the landmass‐level (between England and Continental Europe) and also at the level of the DIYABC pools used (described below). In the latter case, hierarchical *F*_ST_s were used to validate the population poolings used for the DIYABC as in Pedreschi et al. (2014).

### 2.3 Testing the power of the dataset

In order to test scenarios of colonization confidently, it was imperative to ensure that there was enough power in the dataset to accurately detect differentiation and similarity between populations. To do this, we used POWSIM (Ryman & Palm, 2006), a program that estimates the amount of power and type I error for chi‐squared and Fisher's exact tests of homogeneity of allele frequencies across populations. We focused on testing two major aspects of our data for their effects on power and type I error rate; the number of samples per population, and the magnitude of population divergence detectable (*F*_ST_). The number of samples per population in our study ranged from 4 to 37 and the number of samples per DIYABC pool (see below) ranged from 13 to 88; we therefore tested the power and error rates at sample sizes of 4, 12, 20, 28, 36, 60, and 80. For the magnitude of divergence, we tested cases where *F*_ST_ was 0.001, 0.005, 0.01, 0.05, 0.1, and 0.3. For each combination of sample size and *F*_ST_, 1000 data simulation runs were performed. Power was then measured as 1‐β, where β is the type II error rate. β is calculated from the proportion of chi‐squared or Fisher's exact tests across all simulation runs that identified a significant (*p* < .05) difference in allele frequencies between the simulated subpopulations (see Ryman & Palm, 2006 for more detail).

In the context of population structure analyses, a type II error refers to false acceptance of the null hypothesis that allele frequencies between two populations are not different. Thus 1‐β is the probability of correctly rejecting the null hypothesis and therefore identifying population divergence where it exists. For the purposes of this study, a high type II error rate would result in an under estimation of divergence between *C. carassius* populations. We also estimated the Type I (α) error rate in the data, which would represent cases where the null hypothesis was incorrectly rejected, that is, finding population divergence where none exists. These were tested for by running POWSIM at an *F*_ST_ of 0, and quantifying the proportion of runs where significant allele frequencies were found between simulated subpopulations.

### 2.4 Testing the native status of *Carassius carassius* in England

In order to test our three alternative hypotheses for the colonization of *C. carassius* in England, an Approximate Bayesian Computation (ABC) approach was taken, implemented in the program DIYABC (Cornuet et al., 2014). DIYABC simulates datasets of expected summary statistics (ESS) for user‐defined demographic scenarios (“scenario” is used herein to describe a specific population tree topology together with the parameter distribution priors that were assigned to it). These scenarios were then statistically compared to the actual observed data, allowing us to identify those that are most likely to represent the true history of populations (Cornuet et al., 2008). We then estimated the divergence time between populations based on posterior parameter distributions to provide a likely date for the arrival of *C. carassius* in the UK.

To reduce the number of scenarios to be tested, we grouped populations in DIYABC analyses into pools of populations with shared history, a method also employed by Pedreschi et al. (2014). To inform these poolings, it was first necessary to perform a fine‐scale population structure analysis of the 15 populations used. This was carried out using Discriminant Analyses of Principal Components (DAPC), implemented in the Adegenet R package (Jombart, Devillard, & Balloux, 2010). Bayesian Information Criteria (BIC) scores were used to choose the appropriate number of genetic clusters in the dataset. Spline interpolation (Hazewinkel, 1994) was then used to identify the appropriate number of principal components for use in the subsequent discriminant analysis.

Based on the results of the DAPC analysis, populations were grouped into six pools. Those of similar genetic composition (and therefore very likely to have a shared history) were pooled together (see Section 3). However, if populations from either side of the English Channel shared similar genetic composition, then they were separated across pools, to allow for hypothesis testing.

In total, 56 scenarios were tested: six, 39 and 11 representing hypotheses (1), (2) and (3), respectively (Fig. S1). The number of scenarios for each hypothesis reflects the number and plausibility of the possible population histories for the different hypotheses given the results of the population structure analysis. The discriminating factors between scenarios representing different hypotheses were tree topology and, most importantly, the parameter priors for the divergence times between populations (Fig. S1, Table S1). These divergence time priors were set in order to represent the possible time windows of *C. carassius* introduction under our three hypotheses. To test hypothesis (1)—the natural colonization of *C. carassius* more than 7,800 YBP, the time prior for the oldest split between English and Continental European populations was set to 4,000–10,000 generations (equivalent to 8,000–20,000 YBP, assuming a mean generation time of 2 years (Tarkan, Cucherousset, Zięba, Godard, & Copp, 2010; Fig. S1: scenarios 1–6). To test hypothesis (2) that English *C. carassius* were introduced after the 15th century, the same prior was set to 10–1,000 generations (2–2,000 YBP, scenarios 25–44), which very conservatively encompasses all dates of possible live fish translocations to the UK by humans. Finally, to test hypothesis (3) that some populations were native and some introduced, we used multiple combinations of both native and introduced prior dates (as used in hypotheses (1) and (2) scenarios, respectively) for different population splitting events (scenarios 45–56). In the interests of completeness, we also tested an intermediate time window of 10–2,500 generations (20–5,000 YBP, scenarios 7–24). Analyses were performed in a sequential manner, whereby a summary statistic datasets million datasets per scenario were first simulated in DIYABC. For all analyses, the single‐sample summary statistics used were the mean and variance of gene diversity across all polymorphic loci and the mean gene diversity across all loci. The two‐sample summary statistics used were mean and variance of *F*_ST_ and Nei's distance for loci with *F*_ST_ greater than zero between two samples and the mean *F*_ST_ and Nei's distance for all loci. For scenarios including admixture events, the maximum‐likelihood estimates of admixture proportions were also used. See Cornuet et al. (2014) for the exact equations used and their implementation in DIYABC. Finally, the mutation rate (μ) for each locus was given a prior of 1 × 10^−5^–1 × 10^−2^ using a stepwise mutation model allowing for single nucleotide insertions (SNIs).

To reduce computation time, simulated datasets were grouped according to the hypothesis they represented (i.e., (1), (2), or (3)) and these groups were separately compared to the observed data using both approaches offered in DIYABC, logistic regression and “direct estimate.” The latter of which is a count of the number of times that a given scenario simulates one of the closest datasets to the real dataset (Cornuet et al., 2008). The resulting posterior probabilities were used to identify the top two most likely scenarios for each hypothesis (six in total). These were then used in a final test, again using logistic regression and direct estimate, to identify the single most likely scenario of the final six. Model checking analyses, which measures the discrepancy between the model parameter posterior combination and the actual data (Cornuet, Ravigne, & Estoup, 2010), were then carried out to test the robustness of scenario choice. Finally, posterior parameter distributions for effective population size, divergence times, and bottleneck parameters were estimated on the basis of the most likely scenario.

## 3 Results

### 3.1 Microsatellite data analyses

Of the 13 microsatellite loci used, three of the species‐diagnostic loci were monomorphic for *C. carassius*‐specific alleles in the populations studied here (Fig. S2). In all loci, Microchecker showed no consistent signs of null alleles, allele dropout or LD between locus pairs, and although observed locus heterozygosity was generally higher than the expected, tests of Hardy‐Weinberg proportions did not identify any loci that significantly deviated from HWE (Fig. S2).

Within populations, mean observed heterozygosity (across all loci within a population) ranged from 0.03 (GBR2) to 0.4 (GER2). A_r_ ranged from 1.26 (GBR4) to 2.37 (GER2) and correlated with *H*_o_ (adjusted *r* ^2^ = 0.543, *p* = 0.001). *F*_IS_ showed mild inbreeding in several British populations (GBR3, GBR4, GBR8, GBR9) a Belgian population (BEL3) and the German population (GER2) but randomization tests showed that this was only significant in BEL3. Signs of significant outbreeding were observed in three British populations (GBR1, GBR2, GBR7) and were significant in GBR1 and GBR2, although sample sizes in these two populations are small and may not represent the true level of heterozygosity in the population (Table 1).

### 3.2 Statistical power of the dataset

POWSIM analyses showed that the power in the dataset was generally high. At *F*_ST_ ≥ 0.05, sample sizes as low as 4 still resulted in more than 80% probability of correctly identifying true population divergence (Fig. S3). At sample sizes and *F*_ST_ values higher than these, power to detect divergence was above 99%. At populations with sample sizes of less than 10 per population (four populations, see Table S2), power to detect very low levels of population divergence (*F*_ST_ ≤ 0.01) was below 80%. However, when populations of similar genetic composition were pooled for DIYABC analyses, the pool sizes ranged from 13 to 88 (mean = 42.8). The smallest of these pools (UK3, *N* = 13) still had over 80% chance of finding population structure as low as *F*_ST_ = 0.01. Thus, although several individual populations in this dataset suffer from low sample number, population pools used for DIYABC analyses contained high levels of power for accurately detecting even subtle structure between them.

For all sample sizes, the false‐positive rate was less than 5%, meaning that the chance of overestimating divergence times between populations is extremely low.

### 3.3 Population structure in England, Belgium, and Germany

Pairwise *F*_ST_ among populations showed that structure was weakest (*F*_ST_ = 0.0) between the two Belgian populations BEL2 and BEL3, strongest (*F*_ST_ = 0.736) between GBR2 and GBR4 (Fig. S2) and followed a weak IBD pattern, being significantly associated with geographic distance (adjusted *r* = .248, *p* < .001, Fig. S4). Population differentiation was found to be significant in all pairwise comparisons when using the G tests for homogeneity of allele frequencies between populations. However, for some population pairs, *F*_ST_ values were found to be nonsignificant when using permutation tests or Bonferroni corrections, despite being moderate or high in magnitude. Such a result is indicative of a lack of power for calculating *F*_ST_ in these comparisons, and in line with the predictions of the power analyses, these comparisons were indeed those where the number of samples in both populations was low (i.e., <10, Table S2). Importantly however, when pooling populations for the DIYABC analyses, all pairwise *F*_ST_s between pools were highly significant (Table S3) and well above the sample number thresholds indicated by the power analyses for detecting subtle population structure. Hierarchical assessment of population structure using Hierfstat confirmed this and supported the population groupings; with the pools accounting for a large amount of the genetic variation between individuals (*F*_pools_ = 0.244, *p* *=* .001) with the remaining within‐pool variation being low, though still significant (*F*_Ind/pools_ = 0.142, *p* = .001). Population and country assignments also explained significant amounts of the genetic variation (*F*_pop_ *=* 0.36, *p = *.001; *F*_country_ = 0.154, *p = *.001); interestingly however, the landmass to which an individual was assigned (Continental Europe or England) explained none of the variation between individuals (*F*_landmass_ = –0.04, *p = *.482). Taken together, these results suggest considerable shared history between populations in England and Continental Europe and support the use of these poolings as a summary of the relationships between populations.

In the DAPC analysis of population structure, ten genetic clusters were indicated by BIC scores (Fig. S5c). The resulting population‐cluster identities were complex (Fig. S5b), with most populations containing many closely related clusters (Fig. S5a). This made it difficult to identify sets of closely related populations for pooling. Therefore, in order to reliably inform our DIYABC poolings, we incrementally dropped the number of clusters to four which better reflected the large scale patterns of genetic differentiation. Seven principal components and two linear discriminants were retained in this final, four‐cluster DAPC analysis (Figure 1). The resulting inferred population structure showed that many of the English populations showed higher similarities to Continental populations than to neighboring English populations. For example, GBR1 and GBR2 were extremely similar to Belgian populations, and GBR3, GBR6, GBR 7, GBR8, and GBR11 were more similar to populations in northern Germany (Table 1, Figure 1). However, GBR4, GBR5, GBR9, GBR10, all in north Norfolk (eastern England), showed some distinctiveness from Continental populations.

### 3.4 Testing the native status of *Carassius carassius* in England

For the DIYABC analyses, populations were grouped into six pools on the basis of the above DAPC results (population structure and poolings shown in Figure 1). In most cases, it was clear which populations were most similar to each other and thus, how pools should be chosen. However, DAPC results showed complex structure among a subset of UK populations (GBR3, GBR6, GBR7, GBR8, and GBR11, Figure 1), indicative of recent stocking in this area. It was clear that GBR6 was distinct in this group, and so this population was separated and included in the DIYABC analyses as a separate pool. However, the remaining four populations in this group all shared varying proportions of the genetic clusters identified. As analysis these populations separately in DIYABC would have resulted in a prohibitively large number of scenarios, these populations were grouped together in a single pool. Pooling these populations together will likely reduce the accuracy of demographic reconstruction in this region; however, it will have no impact on the reconstruction of the split between UK and continental European populations, which is the primary question in this study.

**Figure 2:**
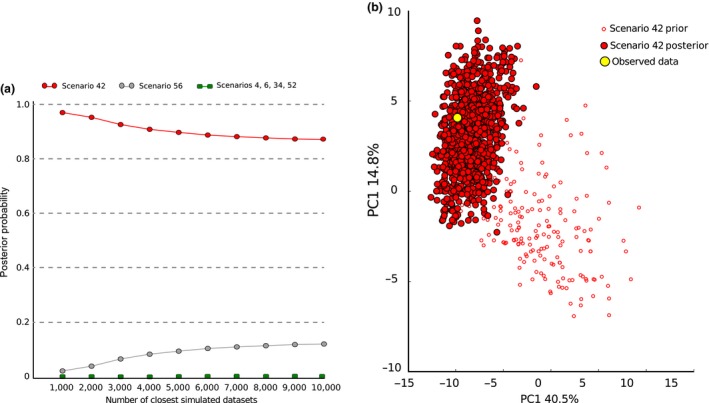
DIYABC comparisons between scenarios. (a) Posterior probabilities that each of the of the six most likely DIYABC scenarios explains the distribution of diversity in the northwest European *Carassius carassius*, calculated using linear regression between the observed dataset and the closest 6,000 simulated datasets; (b) the results of Model Checking of the most likely scenario identified in DIYABC. Note that Observed dataset lies well within the cloud of the predictive posterior parameter distribution

Within‐hypothesis logistic regressions of simulated vs. observed data, performed in DIYABC, showed that the two most likely scenarios for each hypothesis were scenarios 4 and 6 for hypothesis (1), 42 and 34 for hypothesis (2) and 52 and 56 for hypothesis (3). These final six scenarios were then tested against each other, again using logistic regression to find the single most likely scenario of all 56 tested. Scenario 42, representing hypothesis (2), produced datasets that were, by far, the closest to the real data, with a posterior probability of 0.91 (Figure 2a).

**Figure 3:**
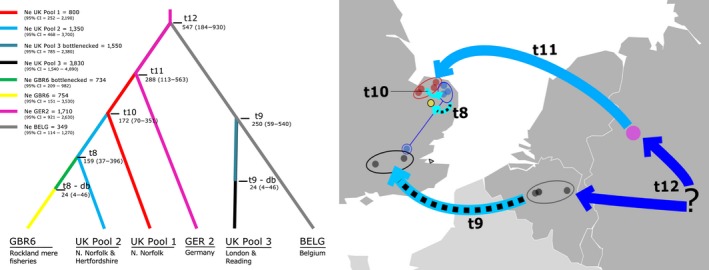
Schematic and map of the most likely scenario for the colonization of the UK by *Carassius carassius* showing two separate introductions of *C. carassius*, approximately 288 and 250 years ago, well after the loss of the Doggerland land bridge. Times are given in years in the schematic and correspond to those inferred by the posterior parameter distributions of DIYABC scenario 42. *t* = time, db = duration of bottleneck event. In the map, time has been coded into the blue color channel used for the arrows, showing older events in dark blue and more recent events in light blue, and bottleneck events are shown by dashed arrows

Scenario 42 (Figure 3) had prior constraints on the split between English and Continental populations (t11) of 10–1,000 generations and, thus, supports a human introduction of *C. carassius* into southeast England <2,000 YBP. Under this scenario, the oldest demographic event (as inferred from the posterior parameter distributions) was the split between German and Belgian populations approximately 547 generations ago (1,094 YBP). However, the most important demographic event for the purposes of testing our hypotheses is the split between English populations (pools UK1, UK2, and RM) and Continental populations (pools GER2 and BELG), at time “t11” in Scenario 42 (Figure 3). Furthermore, this scenario suggests that the ancestral source population of the initial English introduction was more closely related to the German than the Belgian populations sampled here. The date of this English/Continental population split is estimated at 288 generations ago (95% CI = 113–563, Table S4), which corresponds to 576 YBP (95% CI = 226–1,126), *circa* 7,400 years after the loss of the Doggerland land bridge. DIYABC also outputs posterior estimates of population split times scaled by mutation rate and effective population size. The estimated time for the English/Continental population split, scaled by mutation rate estimated by the model, was t11 (μ + SNI) = 9.83 × 10^−2^ (where μ + SNI is the median estimate of the microsatellite mutation rate using the generalized stepwise mutation model (1.11 × 10^−4^ mutations/locus/generation) and SNI is the single nucleotide insertion rate (6.18 × 10^−8^/mutations/locus/generation, Table S4). The median estimate of this mutation rate (μ = 1.11 × 10^−4^/locus/generation), although slow, is still within the realms of that observed in the closely related *C. carpio* (mean = 5.56 × 10^−4^ mutations/locus/generation, 95% CI = 1.52 × 10^−4^–1.63 × 10^−3^, (Yue, David, & Orban, 2007)) and indeed in humans (Ellegren, 2004).

To validate the choice of this scenario as the most likely, we first tested the “goodness‐of‐fit” of Scenario 42 simulated datasets to the real data using statistical model checking (as implemented in DIYABC). This showed that the observed data fell well within the predictive posterior parameter distribution of the simulated data (Figure 2b). Secondly, we calculated the oldest possible date of the English/Continental population split using its upper 95% confidence value under Scenario 42 (563 generations), and assumed the unrealistic, but sometimes possible generation time of 5 years (Tarkan et al., 2010). Despite these extremely conservative values, the split between English and Continental populations was still estimated at 2,815 YBP, *circa* 5,000 years after the flooding of Doggerland. Finally, we inferred t11 (the English/Continental population split) of scenario 42 using the scaled parameter estimate, t11 (μ + SNI). This gave an estimate of 885 generations, or 1,770 years (with a two‐year generation time), which, although older than the un‐scaled estimate, is still over 6,000 years later than the possible natural colonization window. In fact, in order for the scaled estimate to fit the hypothesis of natural colonization (more than 8,000 years ago), assuming a two‐year generation time, the mutation rate would have to be approximately 1.0 × 10^−5^ mutations/locus/generation, at least one order of magnitude lower than reported for microsatellite loci.

Further population splits have occurred more recently from this initial introduction, and there is also support for a second independent introduction of *C. carassius* into the UK (t9) approximately 250 (95% CI = 59–540) generations or 500 (95% CI = 118–1,080) years ago (UK pool 3), from a source population closely related to the Belgian populations sampled here.

## 4 Discussion

### 4.1 Non‐native origins of *Carassius carassius* in England

The primary aim of the present study was to test the contentious assumption that *C. carassius* arrived in southeast England naturally. Of the 56 colonization scenarios tested, one was clearly identified as being the most likely, which inferred that the oldest possible date for the arrival of *C. carassius* in England was *circa* 1126 YBP, almost 7000 years after the loss of the Doggerland land bridge. No scenario with an introduction time consistent with the natural colonization of England received support. This result implies that the only evidence that previously suggested that *C. carassius* were native to England, the pharyngeal bone found at a Roman excavation site (Jones, 1978; Lever, 1977), most likely originates from fish products (e.g., Locker, 2007) rather than from live fish or that *C. carassius* were introduced but did not establish themselves in the wild.

As this result could have important implications for the conservation of *C. carassius* in the UK, we performed rigorous results checking. However, only with highly unrealistic generation times and a mutation rate an order of magnitude slower than that estimated here (and elsewhere, e.g., in *C. carpio* (Yue et al., 2007), mice (Dallas, 1992), sheep (Crawford & Cuthbertson, 1996) and humans (Ellegren, 2004)) would the time for this split support a natural colonization of England by *C. carassius*.

In addition to this result, population structure and DIYABC suggest that there have, in fact, been multiple independent colonization events or introductions into England. For example, in the most likely scenario identified by DIYABC, populations GBR1 and GBR2 split from Belgian populations more recently than they did from other English populations (Figure 3). Indeed, these populations are known to be managed and therefore have likely been stocked in the recent past; GBR1 being a conservation pond, and GBR2 a fish farm. Based on these results it is likely that these fish came from recently imported stocks closely related to the sampled Belgian populations. In contrast to GBR1 and GBR2, DIYABC analyses suggest that all north Norfolk and Hertfordshire populations share a most recent common ancestor with the sampled German population; indicative of a separate introduction.

The most likely date estimated by DIYABC analyses for the first introduction of *C. carassius* in England was 576 YBP (Populations GBR4, 5, 9, 10). This predates the first mention of the species in the literature in the mid‐1700s (Pennant, 1766). However, it does fall perfectly in line with the first known records of *C. carpio* introductions in England, which were imported by 15th century monks for food in monasteries (Lever, 1977). If our estimated date of introduction is accurate, then it is possible that *C. carassius* was also intentionally introduced as a source of food. Indeed, there are mentions of *C. carassius* being used as food in 1778 in Norfolk (Locker, 2007; Woodforde, Winstanley, & Jameson, 2008), and although *C. carassius* does not grow to the size of other carp species, its ability to survive in small, isolated, and often anoxic ponds may have made it an attractive species for use in medieval aquaculture. It is also possible, however, that the introduction of *C. carassius* in England was unintentional. For example, it can be very difficult to tell *C. carassius* and *C. carpio* apart, especially when young and if they are found in sympatry with hybrids present (Wheeler, 2000), as is often the case (Hänfling, Bolton, Harley, & Carvalho, 2005; Sayer et al., 2011). Therefore, stocks of imported *C. carpio* may have also contained some *C. carassius*. Irrespective of the initial motivations however, intentional movements of *C. carassius* especially in north Norfolk have since been common, predominantly for angling purposes (Sayer et al., 2011). This is reflected in the complex population structure found between populations in this region.

It should be noted however that although we are confident that the date of introduction was much earlier than was possible naturally, the confidence intervals around the exact date of introduction are large and there are several factors that could lead to the under or overestimation of this date. Our interpretation of the mechanisms of introduction should therefore be viewed only as a best‐guess. For example, our estimate does not directly pertain to the introduction of English populations, only when they were separated from the sampled Continental European populations. This could have been at the same time as their introduction, but it was more likely prior to their introduction. Thus, it is possible that the arrival time of *C. carassius* in England was even more recent than the DIYABC estimate of population divergence time. Conversely, it is possible that we have underestimated the divergence time between England and continental populations as a result of homoplasious mutations, which are known to occur at microsatellite loci. However, homoplasy is likely to play only a small role here; systems most vulnerable to homoplasy have been shown to be those with high mutation rates and large population sizes (Estoup, Jarne, & Cornuet, 2002), neither of which are found in *C. carassius*. Furthermore, the impacts of homoplasy are largely mitigated by the use of a large number of independent loci (Estoup, Tailliez, Cornuet, & Solignac, 1995; Estoup et al., 2002), as in the present study. Nevertheless, a useful extension of this study would be to repeat this analysis with some high density single nucleotide polymorphism (SNP) data, for example, via restriction site associated DNA (RAD) sequencing, to confirm the date estimates from the present analyses.

Although the sampling in this study was not exhaustive, it covered the areas of England previously thought to contain native *C. carassius* populations, in particular Norfolk, which is thought to have been a stronghold for *C. carassius* in the past (Ellis, 1965; Patterson, 1905; Sayer et al., 2011). It is therefore unlikely that there are unsampled populations of *C. carassius* in England that show further divergence from those of Continental Europe. Furthermore, broad‐scale phylogeographic results in Jeffries et al. (2016) show that Belgian and German populations are likely to be the closest relatives of English *C. carassius* in Europe. Regardless, adding currently un‐sampled populations from Continental Europe could only result in a lower estimate of divergence between English and Continental European samples. We are therefore confident that our estimate represents the earliest possible time frame for the first *C. carassius* introductions into England.

One scenario that we cannot rule out however is the possibility that *C. carassius* colonized naturally, but then either went extinct, or were extirpated by or mixed with stocks which were more recently introduced. In the latter case, the small divergence time observed between English and continental populations could also be driven by admixture between native English *C. carassius*, and continental strains. Only dated fossil evidence and perhaps ancient molecular studies would allow for a definitive test of this scenario, but if it was true, the current English *C. carassius* stocks would still not represent native diversity.

### 4.2 The implications for the conservation of *Carassius carassius*

The results of this study strongly support the human‐mediated introduction of *C. carassius* into England. But what does this mean for the conservation of *C. carassius* in England, a country that has one of the few active projects in place for its conservation (Copp & Sayer, 2010)? In light of these results, should England cease efforts to conserve *C. carassius*? There has been a call recently, for a change in the conservation paradigm, moving away from the unfounded assumption that all non‐native species have detrimental impacts on native ecosystems (Davis et al., 2011). Instead, the authors advocate embracing the idea of constantly changing communities, and moving toward impact‐driven conservation, whereby only those species that have been empirically shown to be invasive and detrimental to native ecosystems and economies are actively managed. Indeed, only a small proportion of freshwater fish introductions have been shown to have detrimental impacts on the native ecosystem, whereas many provide significant ecological and economic benefits (Gozlan, 2008; Schlaepfer, Sax, & Olden, 2011), and sometimes replace ecosystem services lost in extinct species (Schlaepfer et al., 2011). Currently, *C. carassius* could not be labeled as invasive in England, as they are not expanding, in fact, the species is declining throughout its English range (Sayer et al., 2011). With regard to their impact on native ecosystems, to date there has been no attempt to assess this due to the assumption that they were native, however, available studies show that *C. carassius* are widely associated with species‐rich, macrophyte‐dominated ponds (Sayer et al., 2011), which are extremely important ecosystems for conservation (Oertli, Joye, Castella, Cambin, & Lachavanne, 2002). There is no evidence that *C. carassius* negatively impact these habitats, unlike *C. carpio* (Miller & Crowl, 2006), and despite concerns that *C. carassius* may impact the threatened great crested newt (*Triturus cristatus,* Laurenti 1768), this does not seem to be the case in UK ponds, with *C. carassius* often co‐existing with recruiting *T. cristatus* populations (Chan, 2010).

So what of the current conservation efforts for *C. carassius* in England? Perhaps the most important consideration is its threatened status in much of its native European range. Copp et al. (2005) pose the question: Should we treat all introduced species in the same way, even if one such species is endangered in its native range? Indeed, if the goal of conservation science is to protect and enhance biodiversity, it would seem counterproductive to abandon the conservation of *C. carassius* populations in one region when they are threatened in another. The Europe‐wide population structure results in Jeffries et al. (2016) show that English populations, along with those in Belgium and Germany, comprise a distinct part of the overall diversity of *C. carassius* in Europe. And this is made all the more important by the expansion of *C. gibelio* through Europe, especially into the Baltic Sea basin from the south (Deinhardt, 2013; Wouters, Janson, Lusková, & Olsén, 2012). Although the invasive *C. auratus* is present and poses a threat to *C. carassius* in England (as it does in Continental Europe), *C. gibelio* is not yet present and therefore England, with its benign climate that favors *C. carassius* growth and reproduction (Tarkan et al., 2016), represents an important refuge from this threat from invasive Asian congeners.

A last motivation for continuing the conservation of *C. carassius* is their status as an English heritage species. *C. carassius* is affectionately regarded by the zoological and angling communities of England and, as such, has regularly featured in the writings of both groups over the past three centuries—see examples in (Rolfe, 2010), for example, pp. 50–64. Therefore, although our results indicate that *C. carassius* is probably not a native species in the true sense, the species has been an important part of the cultural landscape in England for at least 500 years.

### 4.3 Native or introduced? Testing the status of species

The ability to discriminate between natural and human‐mediated introductions is crucial for determining species status and for conservation plans, but these processes are notoriously difficult to tell apart. The UK, however, presents a rare opportunity to do so, as the loss of the Doggerland land bridge gives us a clear window of possible natural colonization time for freshwater fish and terrestrial species. With newly‐developed population genetics and computational approaches, we have shown here that it is now possible to empirically test the various distinct hypotheses for the mode of species introductions in the UK. In the present study, we focused on *C. carassius*, however another candidate for such a test might be northern pike, *Esox lucius*. Several sources suggest that *E. lucius* may have been originally introduced by humans to Ireland (Ensing, 2014) and there are also examples of pike being introduced with carp species, for example, in Norway (Kleiven, 2013), suggesting a possible link between introductions of the two species. More broadly, the approach described here could be applied to any UK species for which natural colonization was dependent on the land bridge, and to any region or scale where a clear window exists for the natural colonization of a given species. With the falling costs of next‐generation sequencing, the resulting ability to generate huge amounts of genetic data for non‐model species and the continued development of computational approaches, the power of this approach can only increase.

## 5 Conclusions

In the present study, we have shown that, despite the current consensus, *C. carassius* has most likely been introduced in England by humans and would therefore be classified as non‐native. But as its range is contracting, not expanding, it certainly cannot be viewed as invasive. Strong arguments can be made for the continued conservation of *C. carassius* in England; however, there is a need for studies that assess the ecological role that the species plays in England, to ensure that no true native species are imperiled by its presence.

Beyond this specific case, our results bring to light much broader and timely questions in invasion and conservation biology, which are as follows: how many assumptions about the native status of other freshwater (or, indeed, terrestrial) species in the UK would stand up to the same tests as performed here if the data were available to perform it? And what do we do about it if they don't? The approach used in this study allows us to address the first of these questions, but the second remains the subject of hot debate.

## Conflict of Interest

None declared.
