# Supplementary material for: Genetic evidence challenges the native status of a threatened freshwater fish (Carassius carassius) in England
Source: Ecol Evol. 2017 Mar 22;7(9):2871–82. doi: 10.1002/ece3.2831 (PMC5415527; doi:10.1002/ece3.2831)
Supplement: Supplementary file 1 [file ECE3-7-2871-s001.doc]

# Genetic evidence challenges the native status of a threatened freshwater fish (*Carassius carassius*) in England

**Authors:** Daniel L Jeffries^1,2^, Gordon H Copp^2^, Gregory Maes^3^, Lori Lawson Handley^1^, Carl D Sayer^4^, Bernd Hänfling^1^

^1^ *Evolutionary Biology Group, School of Biological, Biomedical and Environmental Sciences, Hardy Building, University of Hull, Hull, HU6 7RX, UK*

*^2^Salmon and Freshwater Team, Cefas, Pakeﬁeld Road, Lowestoft, Suffolk NR33 0HT, UK, and Department of Life and Environmental Sciences, Faculty of Science and Technology, Bournemouth University, Poole, UK*

*^3^* *Laboratory of Biodiversity and Evolutionary Genomics, University of Leuven, B-3000 Leuven, Belgium*

^4^ *Pond Restoratation Research Group, Environmental Change Research Centre, Department of Geography, University College London, Pearson Building, Gower Street, London, WC1E 6BT, UK*

**Supplementary Materials**

Table S1. Prior parameters for all scenarios used in DIYABC analyses for *C. carassius*.

| Hypothesis tested | Parameter | Defined Prior Distribution | Times in Years  (*t* x 2) | Conditions |
| --- | --- | --- | --- | --- |
| All | *N1–N6* | Uniform  [10–5000] |  |  |
|  | *ra* | Uniform  [0.001– 0.999] |  |  |
|  | *db* | Uniform  [1–100] | 2–500 |  |
| i) | *t1* | Uniform  [10–10000] | 20–20000 | *< t2, t3, t4* |
|  | *t2* | Uniform  [10–10000] | 20–20000 | *< t3, t4* |
|  | *t3* | Uniform  [10–10000] | 20–20000 | *< t4* |
|  | *t4* | Uniform  [4000–10000] | 8000–20000 |  |
| ii) | *t5* | Uniform  [10- 2500] | 20–5000 | *<t6, t7, t8* |
|  | *t6* | Uniform  [10–2500] | 20–5000 | *< t7, t8* |
|  | *t7* | Uniform  [10–2500] | 20–5000 | *< t8* |
|  | *t8* | Uniform  [10–2500] | 20–5000 |  |
|  | *t9* | Uniform  [10–1000] | 20–2000 | *<t10, t11, t12* |
|  | *t9b* | Uniform  [10–1000] | 20–2000 | *<t10, t11, t12* |
|  | *t10* | Uniform  [10–1000] | 20–2000 | *< t11, t12* |
|  | *t11* | Uniform  [10–1000] | 20–2000 | *< t12* |
|  | *t12* | Uniform  [10­–­1000] | 20–2000 |  |
| iii) | *t12a* | Uniform  [10–2500] | 20–5000 | *<t13, t14, t15, t16* |
|  | *t13* | Uniform  [10–2500] | 20–5000 | *< t14, t15, t16* |
|  | *t14* | Uniform  [10–2500] | 20–5000 | *< t15, t16* |
|  | *t15* | Uniform  [10–2500] | 20–5000 | *< t16* |
|  | *t16* | Uniform  [4000–10000] | 8000–20000 |  |
|  | *t17* | Uniform  [10–1000] | 20–2000 | *<t18, t19, t20* |
|  | *t18* | Uniform  [10–1000] | 20–2000 | *< t19, t20* |
|  | *t19* | Uniform  [10–1000] | 20–2000 | *< t20* |
|  | *t20* | Uniform  [4000–10000] | 8000–20000 |  |

Table S2. Pair-wise *F*_ST_s for each population studied (top semi-matrix). Values in red are those where a moderate or high *F*st (> 0.2) did not show significance using either P-values or confidence intervals (95%). The bottom semi matrix shows the total number of samples in each pairwise comparison.

| **N samples** |  | 9 | 4 | 27 | 13 | 12 | 8 | 21 | 24 | 7 | 37 | 27 | 20 | | | 14 | 20 | 14 |
| --- | --- | --- | --- | --- | --- | --- | --- | --- | --- | --- | --- | --- | --- | --- | --- | --- | --- | --- |
|  | **Population** | GBR1 | GBR2 | GBR4 | BEL1 | BEL2 | BEL3 | GER2 | GBR7 | GBR3 | GBR8 | GBR9 | GBR11 | | | GBR5 | GBR6 | GBR10 |
| 9 | GBR1 |  | 0.3148 | ***0.6323*** | **0.3711** | ***0.207*** | ***0.366*** | ***0.2842*** | ***0.5017*** | *0.3676* | ***0.4369*** | ***0.6114*** | ***0.2975*** | | | ***0.5956*** | ***0.3518*** | ***0.4373*** |
| 4 | GBR2 | 13 |  | ***0.7369*** | **0.353** | *0.2644* | *0.3795* | ***0.3334*** | ***0.6109*** | *0.535* | ***0.5616*** | ***0.7164*** | ***0.3809*** | | | ***0.7*** | ***0.4851*** | *0.5358* |
| 27 | GBR4 | 36 | 31 |  | ***0.6616*** | ***0.494*** | ***0.5857*** | ***0.3076*** | ***0.1943*** | ***0.4229*** | ***0.3497*** | **0.2982** | ***0.2058*** | | | ***0.2351*** | ***0.4976*** | ***0.1883*** |
| 13 | BEL1 | 22 | 17 | 40 |  | 0.0755 | *0.0204* | ***0.269*** | ***0.5601*** | ***0.3835*** | ***0.4882*** | ***0.5886*** | ***0.2985*** | | | ***0.5648*** | ***0.3322*** | ***0.4858*** |
| 12 | BEL2 | 21 | 16 | 39 | 25 |  | 0 | ***0.1878*** | ***0.4225*** | ***0.2504*** | ***0.3869*** | ***0.4159*** | ***0.157*** | | | ***0.3934*** | ***0.2715*** | ***0.3002*** |
| 8 | BEL3 | 17 | 12 | 35 | 21 | 20 |  | ***0.1909*** | ***0.4879*** | ***0.3133*** | ***0.4379*** | ***0.5058*** | ***0.2165*** | | | ***0.4763*** | ***0.299*** | ***0.3754*** |
| 21 | GER2 | 30 | 25 | 48 | 34 | 33 | 29 |  | ***0.1669*** | *0.0682* | ***0.1352*** | ***0.3144*** | ***0.134*** | | | ***0.2451*** | ***0.1502*** | ***0.1845*** |
| 24 | GBR7 | 33 | 28 | 51 | 37 | 36 | 32 | 45 |  | 0.1526 | ***0.0717*** | ***0.3643*** | ***0.164*** | | | ***0.2876*** | ***0.3282*** | ***0.161*** |
| 7 | GBR3 | 16 | 11 | 34 | 20 | 19 | 15 | 28 | 31 |  | 0.0208 | **0.422** | *0.0896* | | | **0.3539** | 0.0795 | *0.2133* |
| 37 | GBR8 | 46 | 41 | 64 | 50 | 49 | 45 | 58 | 61 | 44 |  | ***0.4205*** | ***0.1837*** | | | ***0.3569*** | ***0.2124*** | ***0.2581*** |
| 27 | GBR9 | 36 | 31 | 54 | 40 | 39 | 35 | 48 | 51 | 34 | 64 |  | ***0.2054*** | | | 0.0287 | ***0.4394*** | ***0.2007*** |
| 20 | GBR11 | 29 | 24 | 47 | 33 | 32 | 28 | 41 | 44 | 27 | 57 | 47 |  | | | ***0.1633*** | ***0.2274*** | ***0.1071*** |
| 14 | GBR5 | 23 | 18 | 41 | 27 | 26 | 22 | 35 | 38 | 21 | 51 | 41 | 34 | | |  | ***0.3894*** | ***0.1972*** |
| 20 | GBR6 | 29 | 24 | 47 | 33 | 32 | 28 | 41 | 44 | 27 | 57 | 47 | 40 | | | 34 |  | ***0.318*** |
| 14 | GBR10 | 23 | 18 | 41 | 27 | 26 | 22 | 35 | 38 | 21 | 51 | 41 | 34 | | | 28 | 34 |  |
|  |  |  |  |  |  |  |  |  |  |  |  |  |  | | |  |  |  |
| Fst (WC), P vals from 10500 permutation and Sequential bonferroni correction (significant values in bold), | | | | | | | | | | | | | | |  | | | |
| 95% confidence intervals calculated via 1000 bootstraps. Those not including zero are in italics. | | | | | | | | | | | | | |  | | | | |
|  |  |  |  |  |  |  |  |  |  |  |  |  |  | | |  |  |  |

Table S3. Pair-wise *F*_ST_s among population pools used in DIYABC (top semi-matrix).

| N samples |  | 82 | 88 | 21 | 20 | 13 | 33 |
| --- | --- | --- | --- | --- | --- | --- | --- |
|  | Pool | UK1 | UK2 | FFG | RM | UK3 | BELG |
| 82 | UK1 |  | ***0.24*** | ***0.28*** | ***0.36*** | ***0.47*** | ***0.41*** |
| 88 | UK2 | 170 |  | ***0.18*** | ***0.19*** | ***0.37*** | ***0.32*** |
| 21 | FFG | 103 | 109 |  | ***0.14*** | ***0.30*** | ***0.26*** |
| 20 | RM | 102 | 108 | 41 |  | ***0.36*** | ***0.28*** |
| 13 | UK3 | 95 | 101 | 34 | 33 |  | ***0.23*** |
| 33 | BELG | 115 | 121 | 54 | 53 | 46 |  |

Fst (WC) *P* values from 1500 permutations and Sequential Bonferroni correction (significant values in bold), 95% confidence intervals calculated via 1000 bootstraps. Those not including zero are in italics

Table S4. All posterior parameter distributions for all scenario 42 - identified as the most likely scenario for the colonisation of *C. carassius* into England by DIYABC analyses.

| **Parameter** | **mean** | **median** | **mode** | **q025** | **q050** | **q250** | **q750** | **q950** | **q975** |
| --- | --- | --- | --- | --- | --- | --- | --- | --- | --- |
| **Original** |  |  |  |  |  |  |  |  |  |
| N1 | 9.45E+02 | 8.00E+02 | 5.60E+02 | 2.02E+02 | 2.52E+02 | 5.42E+02 | 1.12E+03 | 2.19E+03 | 2.99E+03 |
| N2 | 1.58E+03 | 1.35E+03 | 1.19E+03 | 3.45E+02 | 4.68E+02 | 8.84E+02 | 2.04E+03 | 3.70E+03 | 4.15E+03 |
| N3 | 1.75E+03 | 1.71E+03 | 1.58E+03 | 8.00E+02 | 9.21E+02 | 1.38E+03 | 2.11E+03 | 2.63E+03 | 2.80E+03 |
| N4 | 1.14E+03 | 7.54E+02 | 3.31E+02 | 1.14E+02 | 1.51E+02 | 4.00E+02 | 1.54E+03 | 3.53E+03 | 4.15E+03 |
| N5 | 3.63E+03 | 3.83E+03 | 4.79E+03 | 1.13E+03 | 1.54E+03 | 2.98E+03 | 4.49E+03 | 4.89E+03 | 4.95E+03 |
| N6 | 4.70E+02 | 3.49E+02 | 3.20E+02 | 9.89E+01 | 1.14E+02 | 2.36E+02 | 5.18E+02 | 1.27E+03 | 1.81E+03 |
| t9 | 2.72E+02 | 2.50E+02 | 2.34E+02 | 4.00E+01 | 5.92E+01 | 1.54E+02 | 3.67E+02 | 5.42E+02 | 6.23E+02 |
| db | 2.43E+01 | 2.43E+01 | 8.67E+00 | 2.04E+00 | 3.60E+00 | 1.22E+01 | 3.62E+01 | 4.61E+01 | 4.73E+01 |
| N5a | 1.67E+02 | 7.89E+01 | 1.88E+01 | 1.19E+01 | 1.41E+01 | 3.54E+01 | 2.03E+02 | 7.03E+02 | 7.97E+02 |
| t9b | 1.79E+02 | 1.59E+02 | 1.05E+02 | 2.54E+01 | 3.73E+01 | 9.58E+01 | 2.39E+02 | 3.96E+02 | 4.51E+02 |
| N4a | 7.81E+02 | 8.49E+02 | 9.69E+02 | 2.47E+02 | 3.48E+02 | 6.77E+02 | 9.44E+02 | 9.89E+02 | 9.96E+02 |
| t10 | 1.88E+02 | 1.72E+02 | 1.43E+02 | 6.01E+01 | 7.08E+01 | 1.25E+02 | 2.32E+02 | 3.51E+02 | 3.94E+02 |
| t11 | 3.06E+02 | 2.88E+02 | 2.68E+02 | 8.08E+01 | 1.13E+02 | 1.99E+02 | 3.83E+02 | 5.63E+02 | 6.76E+02 |
| N1a | 6.52E+02 | 7.17E+02 | 9.82E+02 | 8.10E+01 | 1.42E+02 | 4.61E+02 | 8.84E+02 | 9.78E+02 | 9.86E+02 |
| t12 | 5.52E+02 | 5.47E+02 | 4.37E+02 | 1.46E+02 | 1.84E+02 | 3.72E+02 | 7.38E+02 | 9.30E+02 | 9.53E+02 |
| µmic_1 | 1.17E-04 | 1.11E-04 | 1.00E-04 | 1.00E-04 | 1.00E-04 | 1.04E-04 | 1.22E-04 | 1.57E-04 | 1.72E-04 |
| pmic_1 | 2.86E-01 | 2.95E-01 | 3.00E-01 | 2.21E-01 | 2.42E-01 | 2.80E-01 | 3.00E-01 | 3.00E-01 | 3.00E-01 |
| snimic_1 | 4.00E-07 | 6.18E-08 | 1.00E-08 | 1.02E-08 | 1.08E-08 | 2.16E-08 | 2.59E-07 | 1.95E-06 | 2.99E-06 |
|  |  |  |  |  |  |  |  |  |  |
| **Composite** |  |  |  |  |  |  |  |  |  |
| N1(u+sni)_1 | 5.69E-02 | 9.04E-03 | 9.04E-03 | 9.04E-03 | 9.04E-03 | 9.04E-03 | 9.04E-03 | 9.04E-03 | 1.05E-01 |
| N2(u+sni)_1 | 3.58E-01 | 6.49E-03 | 6.49E-03 | 6.49E-03 | 6.49E-03 | 6.49E-03 | 8.36E-02 | 1.74E+00 | 1.74E+00 |
| N3(u+sni)_1 | 1.30E-01 | 1.99E-03 | 1.99E-03 | 1.99E-03 | 1.99E-03 | 1.99E-03 | 1.38E-02 | 7.29E-01 | 7.29E-01 |
| N4(u+sni)_1 | 1.56E+00 | 2.03E+00 | 2.03E+00 | 3.97E-03 | 3.97E-03 | 1.55E+00 | 2.03E+00 | 2.03E+00 | 2.03E+00 |
| N5(u+sni)_1 | 2.09E+00 | 2.20E+00 | 2.20E+00 | 3.75E-03 | 9.07E-01 | 2.20E+00 | 2.20E+00 | 2.20E+00 | 2.20E+00 |
| N6(u+sni)_1 | 1.98E-02 | 1.53E-03 | 1.53E-03 | 1.53E-03 | 1.53E-03 | 1.53E-03 | 1.53E-03 | 1.53E-03 | 1.53E-03 |
| t9(u+sni)_1 | 8.58E-02 | 6.26E-02 | 6.35E-04 | 6.35E-04 | 6.35E-04 | 6.35E-04 | 1.74E-01 | 1.74E-01 | 1.74E-01 |
| db(u+sni)_1 | 1.13E-03 | 1.04E-04 | 1.04E-04 | 1.04E-04 | 1.04E-04 | 1.04E-04 | 1.04E-04 | 9.59E-03 | 2.05E-02 |
| N5a(u+sni)_1 | 1.55E-01 | 6.24E-02 | 1.15E-03 | 1.15E-03 | 1.15E-03 | 3.59E-03 | 3.58E-01 | 4.03E-01 | 4.03E-01 |
| t9b(u+sni)_1 | 2.83E-02 | 1.42E-03 | 6.20E-04 | 6.20E-04 | 6.20E-04 | 6.27E-04 | 1.73E-02 | 1.97E-01 | 2.11E-01 |
| N4a(u+sni)_1 | 2.85E-02 | 1.55E-03 | 1.55E-03 | 1.55E-03 | 1.55E-03 | 1.55E-03 | 2.05E-03 | 3.35E-01 | 3.82E-01 |
| t10(u+sni)_1 | 3.22E-02 | 1.10E-02 | 1.10E-02 | 1.10E-02 | 1.10E-02 | 1.10E-02 | 1.30E-02 | 2.10E-01 | 2.51E-01 |
| t11(u+sni)_1 | 2.62E-01 | 2.92E-01 | 2.92E-01 | 2.85E-02 | 2.85E-02 | 2.92E-01 | 2.92E-01 | 2.92E-01 | 2.92E-01 |
| N1a(u+sni)_1 | 8.40E-02 | 1.06E-03 | 1.06E-03 | 1.06E-03 | 1.06E-03 | 1.06E-03 | 8.16E-03 | 4.18E-01 | 4.18E-01 |
| t12(u+sni)_1 | 3.11E-01 | 3.75E-01 | 3.75E-01 | 4.41E-02 | 4.66E-02 | 3.13E-01 | 3.75E-01 | 3.75E-01 | 3.75E-01 |
|  |  |  |  |  |  |  |  |  |  |
| **Scaled** |  |  |  |  |  |  |  |  |  |
| N1/Mean(N) | 1.16E+00 | 4.30E-01 | 3.11E-02 | 3.11E-02 | 3.11E-02 | 5.64E-02 | 2.46E+00 | 3.43E+00 | 3.43E+00 |
| N2/Mean(N) | 4.70E-01 | 3.07E-02 | 3.07E-02 | 3.07E-02 | 3.07E-02 | 3.07E-02 | 3.07E-02 | 3.27E+00 | 3.27E+00 |
| N3/Mean(N) | 2.35E-01 | 4.39E-03 | 4.39E-03 | 4.39E-03 | 4.39E-03 | 4.39E-03 | 4.39E-03 | 2.88E+00 | 2.88E+00 |
| N4/Mean(N) | 1.49E+00 | 1.61E-01 | 2.25E-02 | 2.25E-02 | 2.25E-02 | 2.25E-02 | 3.37E+00 | 3.37E+00 | 3.37E+00 |
| N5/Mean(N) | 1.27E-01 | 8.40E-03 | 8.40E-03 | 8.40E-03 | 8.40E-03 | 8.40E-03 | 8.82E-03 | 2.95E-01 | 1.83E+00 |
| N6/Mean(N) | 5.35E-02 | 6.82E-03 | 6.82E-03 | 6.82E-03 | 6.82E-03 | 6.82E-03 | 6.82E-03 | 6.82E-03 | 6.82E-03 |
| t9/Mean(N) | 2.58E-01 | 2.27E-03 | 2.24E-03 | 2.24E-03 | 2.24E-03 | 2.24E-03 | 7.18E-01 | 7.18E-01 | 7.18E-01 |
| db/Mean(N) | 6.09E-03 | 5.00E-04 | 5.00E-04 | 5.00E-04 | 5.00E-04 | 5.00E-04 | 1.05E-03 | 4.72E-02 | 7.57E-02 |
| N5a/Mean(N) | 1.11E+00 | 1.56E+00 | 1.56E+00 | 3.96E-03 | 3.96E-03 | 1.38E-01 | 1.56E+00 | 1.56E+00 | 1.56E+00 |
| t9b/Mean(N) | 4.25E-01 | 6.97E-01 | 6.97E-01 | 2.27E-03 | 2.27E-03 | 2.27E-03 | 6.97E-01 | 6.97E-01 | 6.97E-01 |
| N4a/Mean(N) | 1.54E-01 | 3.73E-03 | 3.73E-03 | 3.73E-03 | 3.73E-03 | 3.73E-03 | 3.73E-03 | 1.57E+00 | 1.57E+00 |
| t10/Mean(N) | 2.06E-01 | 3.38E-02 | 3.20E-02 | 3.20E-02 | 3.20E-02 | 3.20E-02 | 2.78E-01 | 7.87E-01 | 7.87E-01 |
| t11/Mean(N) | 8.10E-01 | 1.08E+00 | 1.08E+00 | 7.54E-02 | 7.54E-02 | 5.39E-01 | 1.08E+00 | 1.08E+00 | 1.08E+00 |
| N1a/Mean(N) | 8.43E-01 | 1.28E+00 | 1.28E+00 | 4.68E-03 | 4.68E-03 | 1.13E-02 | 1.28E+00 | 1.28E+00 | 1.28E+00 |
| t12/Mean(N) | 8.18E-01 | 1.07E+00 | 1.27E+00 | 9.18E-02 | 9.18E-02 | 2.37E-01 | 1.27E+00 | 1.27E+00 | 1.27E+00 |


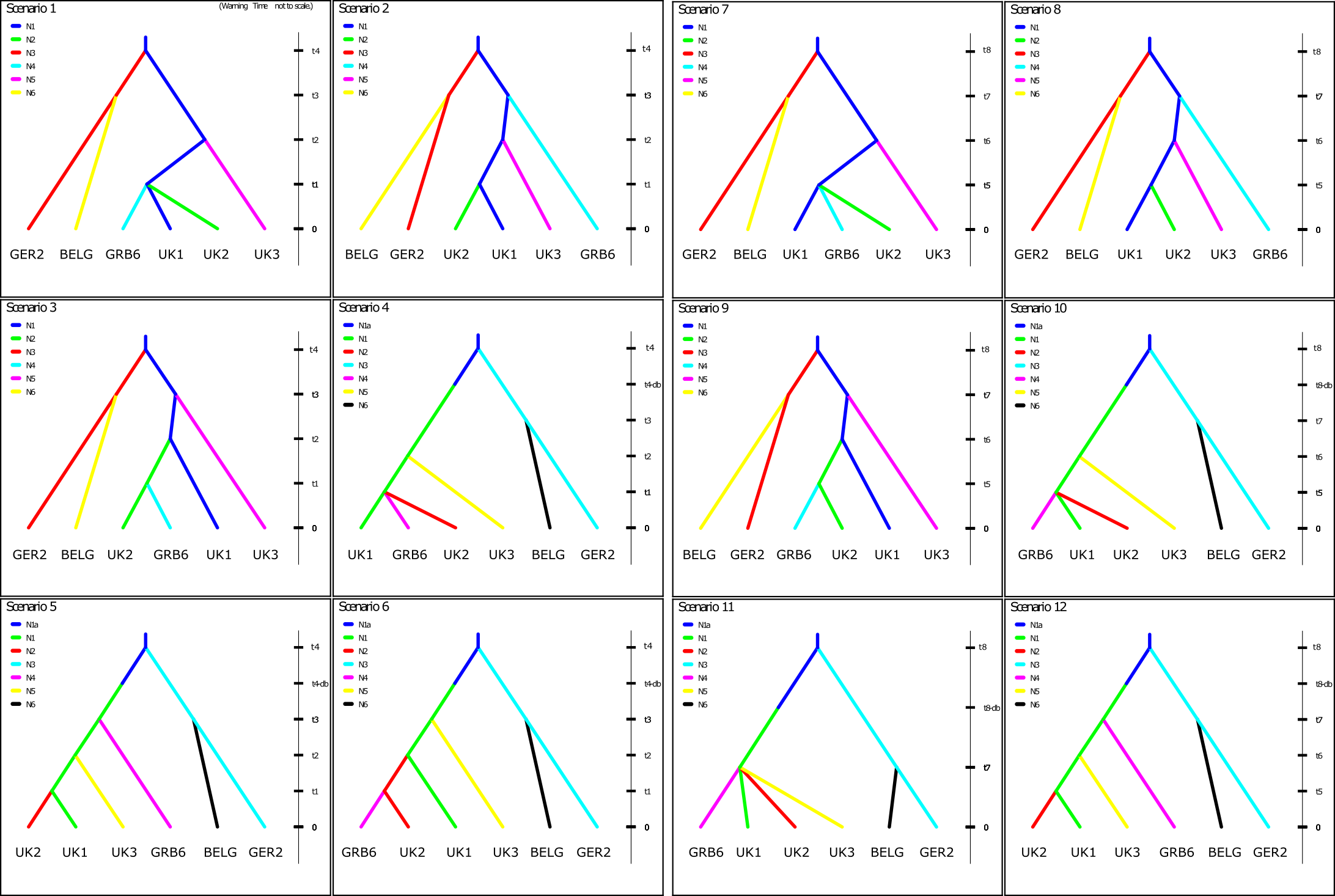


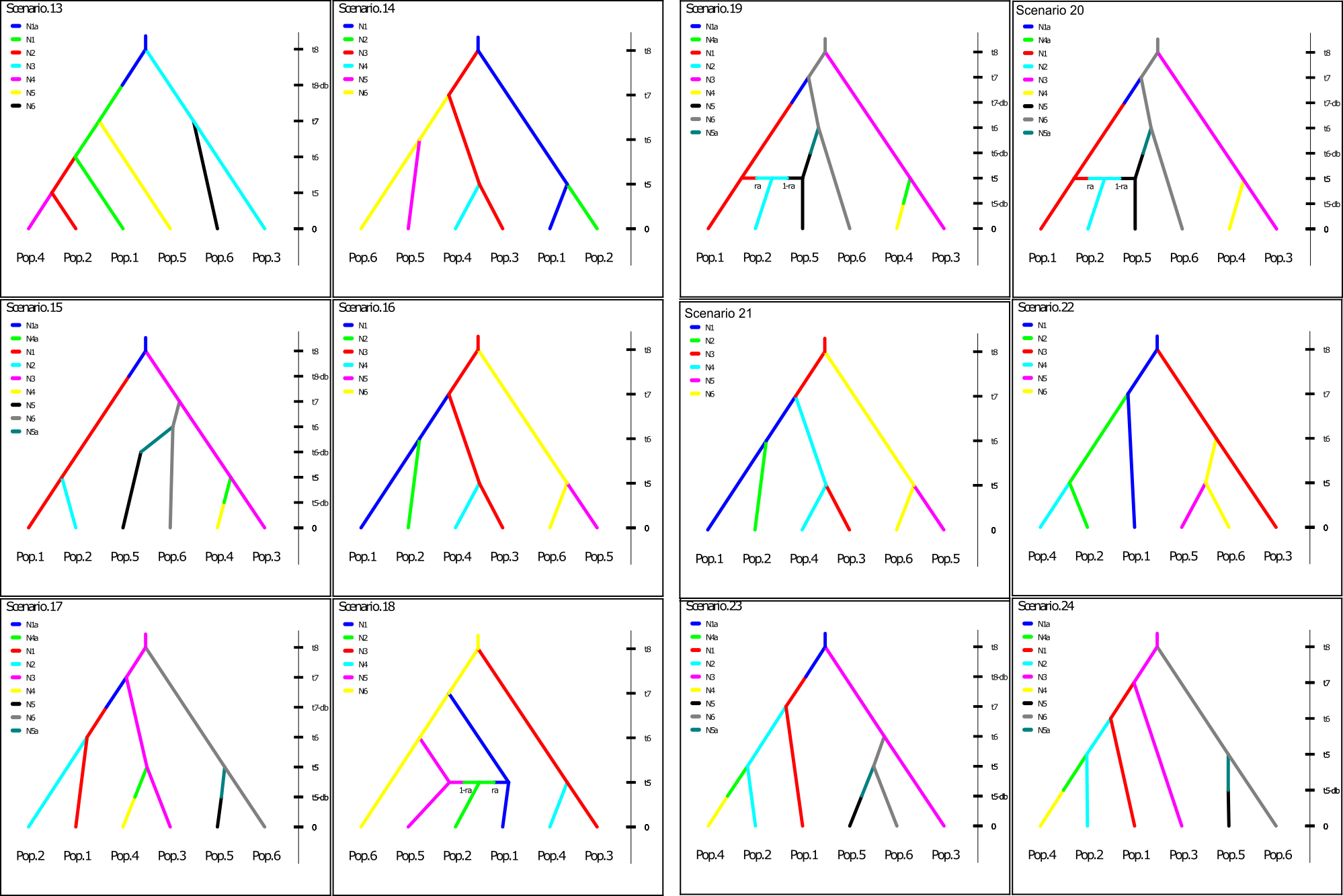


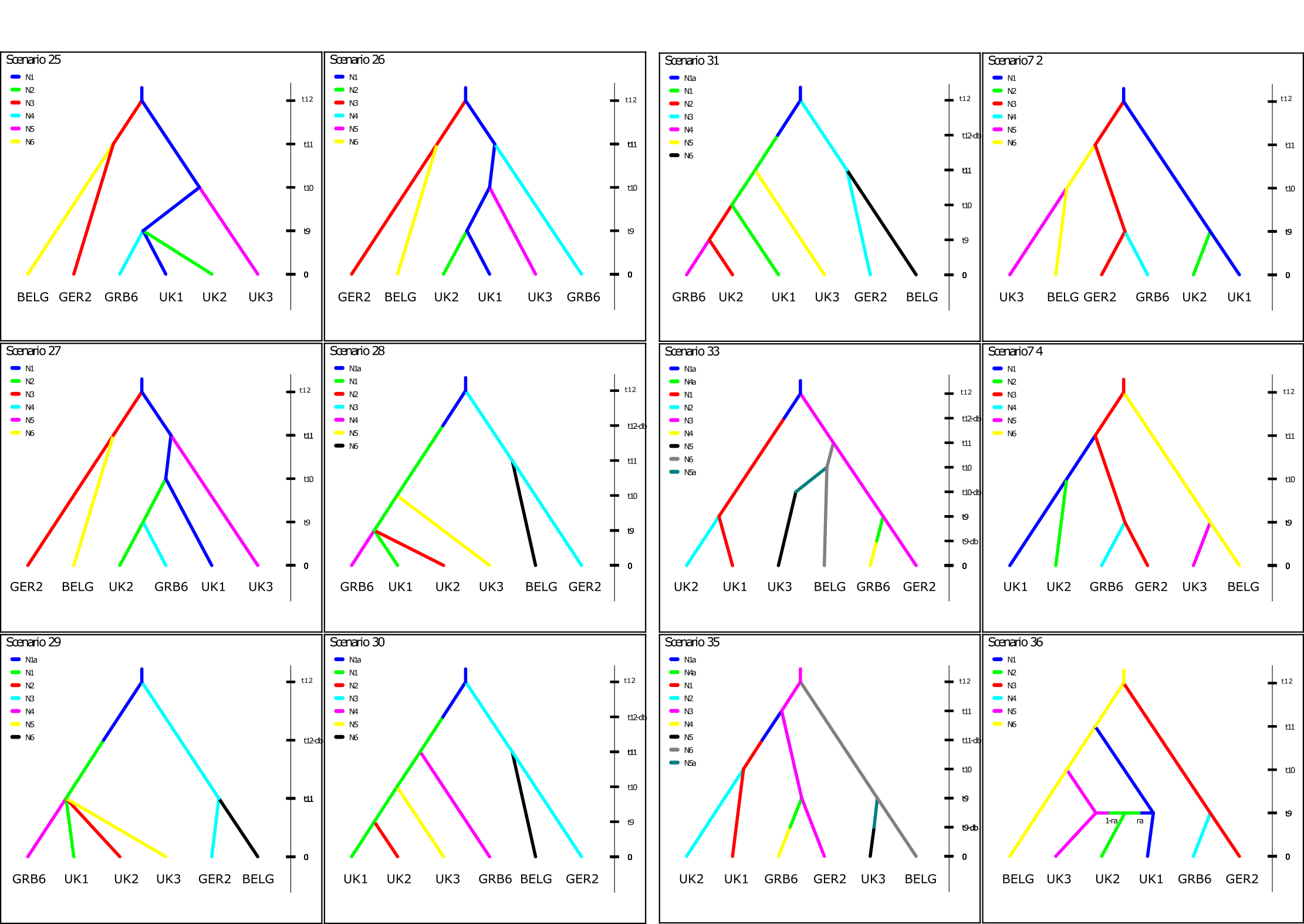


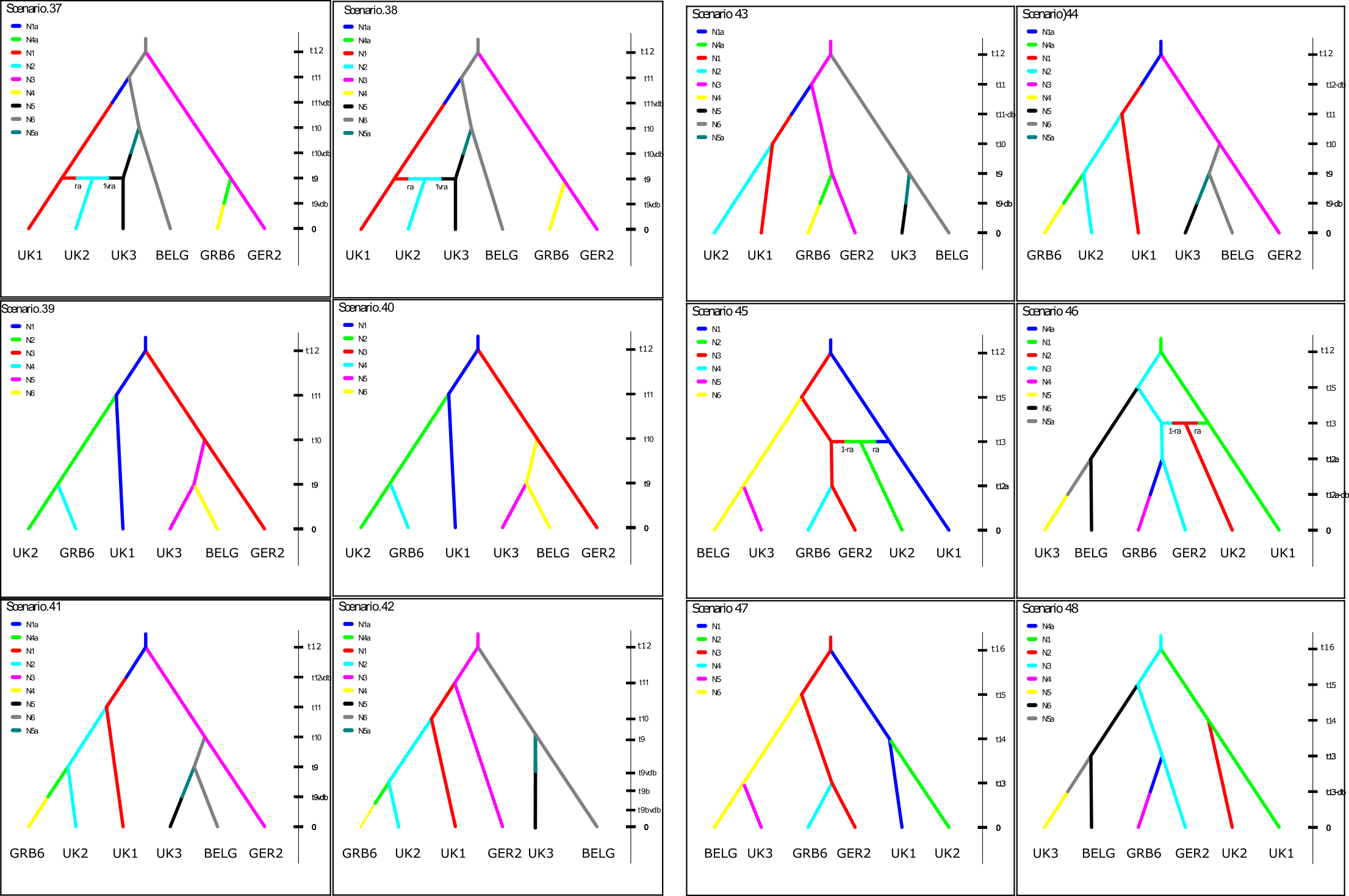


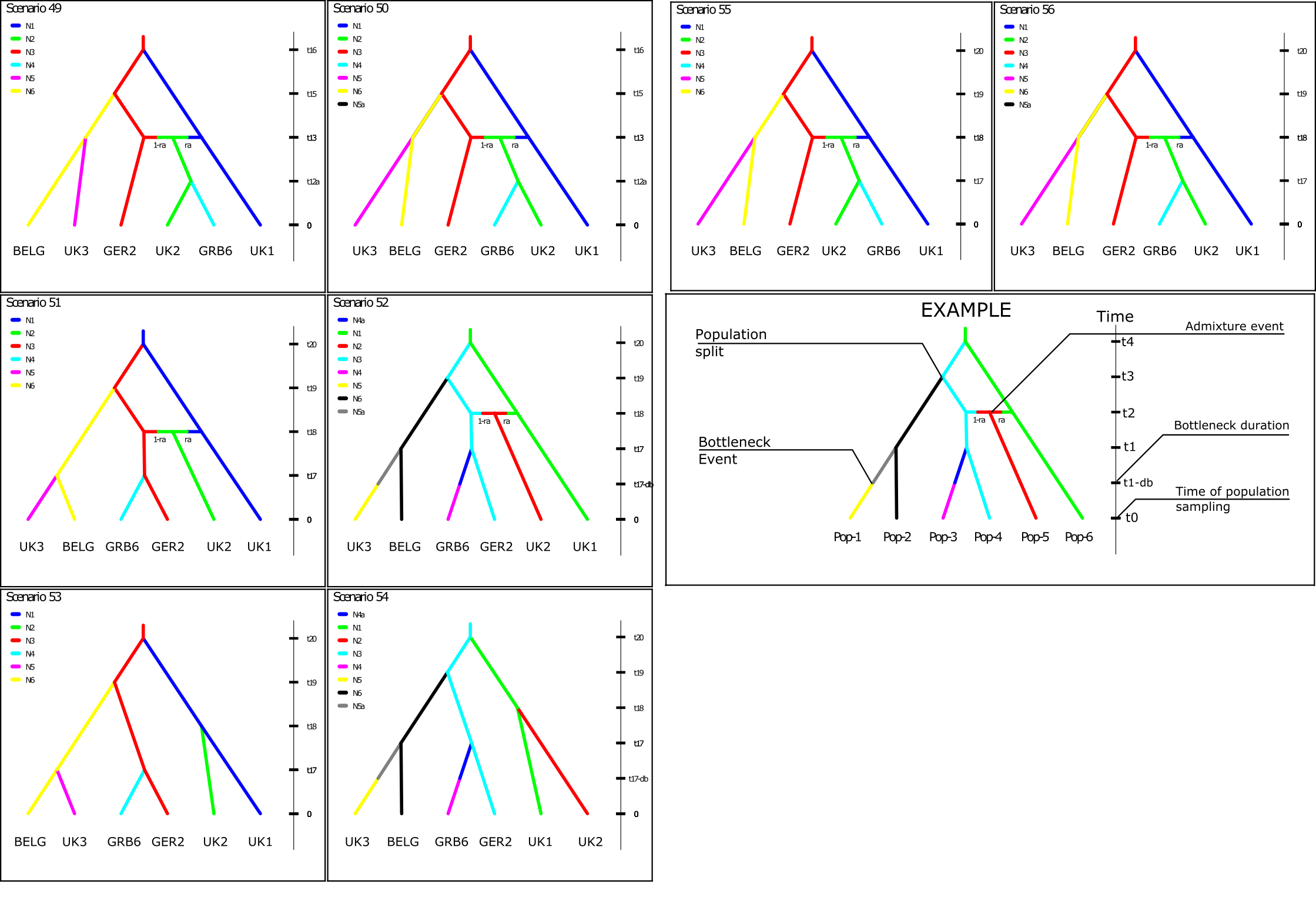


**Figure S1.** All 56 scenarios tested in DIYABC analysis. See EXAMPLE pane for explanation of the schematic. Colours of branches correspond to the effective population size of each population. Colours are not consistent across plots, but are generated randomly by DIYABC. db = the time duration of a bottleneck event, ra and 1-ra are admixture frequencies.


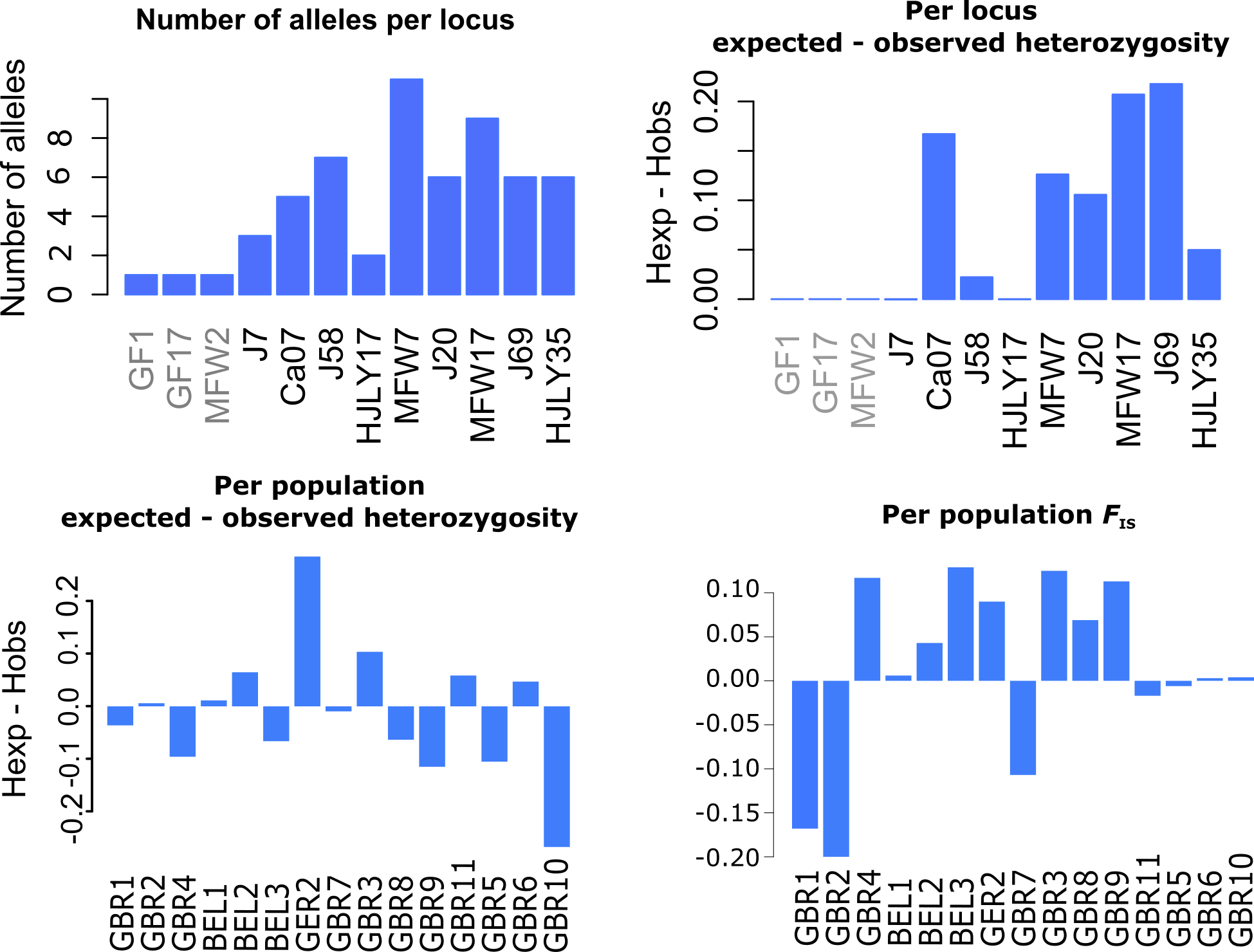


Figure S2. Per locus and population summary statistics.


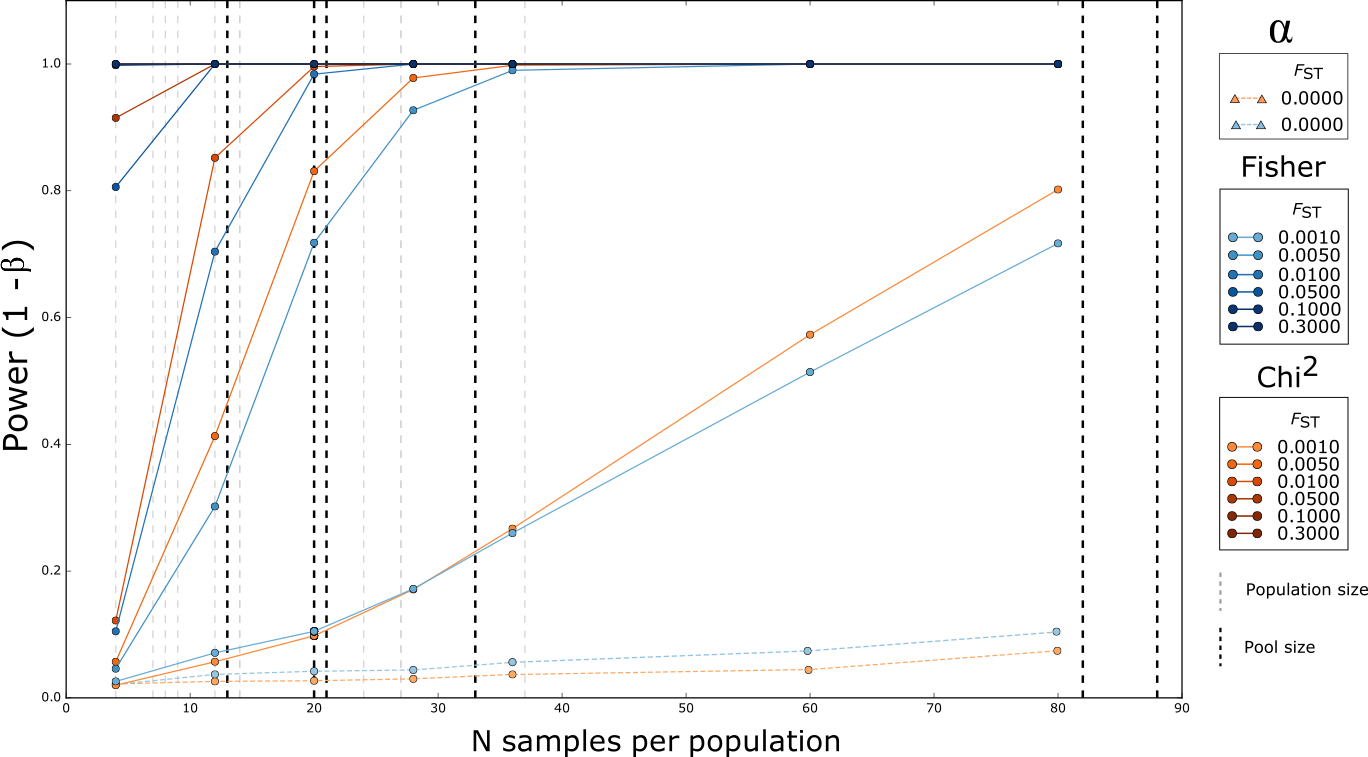


Figure S3. POWSIM analyses of statistical power for correctly identifying population divergence. Power (1-β) was calculated for chi-squared tests and Fisher’s exact tests of homogeneity of allele frequencies among subpopulations for a range of sample sizes and *F*_ST_ values. Type I error rate (α) was calculated from false positive results of allele frequency differences when *F*_ST_ was zero.


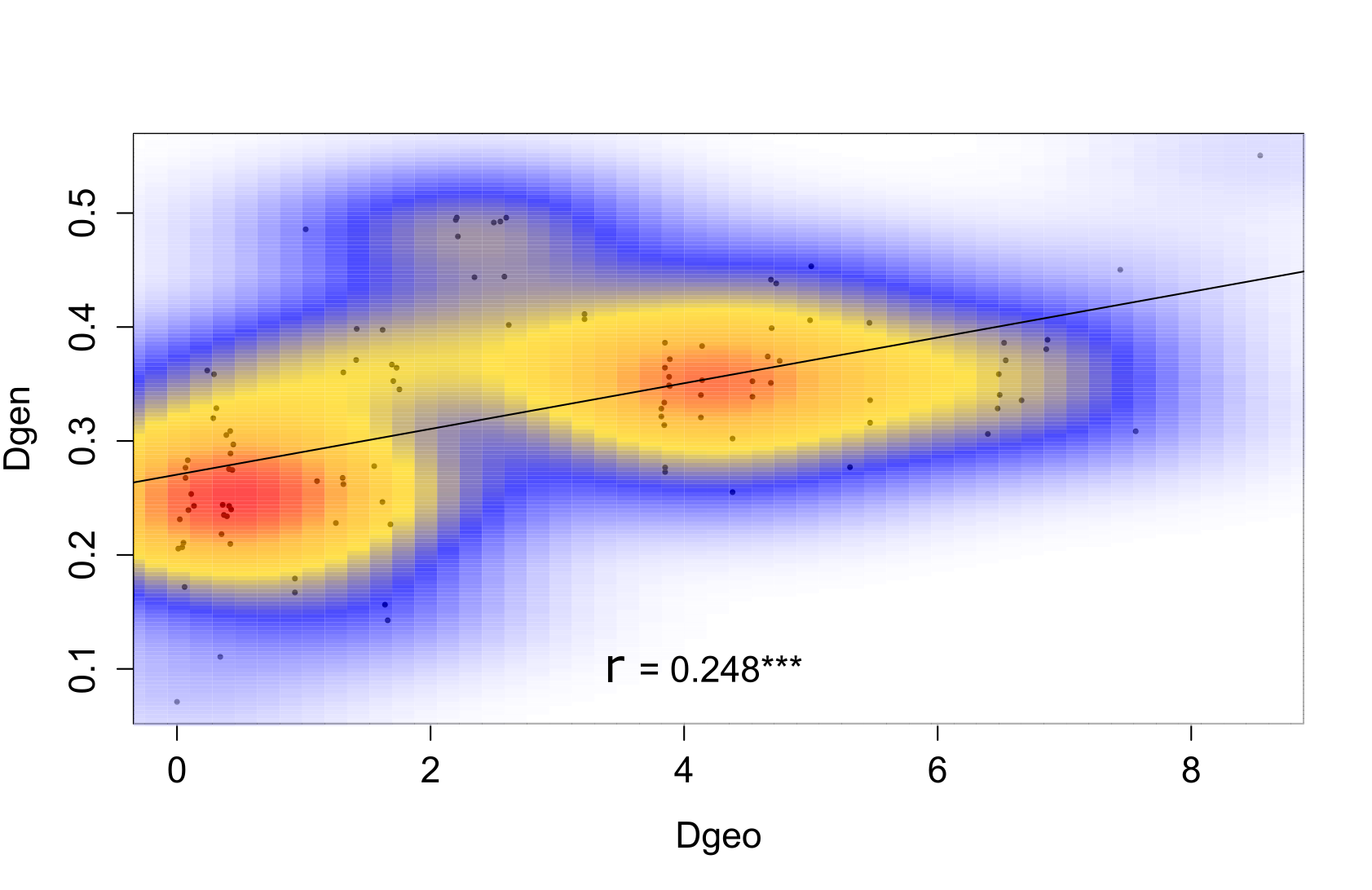


Figure S4. Isolation by distance in the 15 populations sampled.


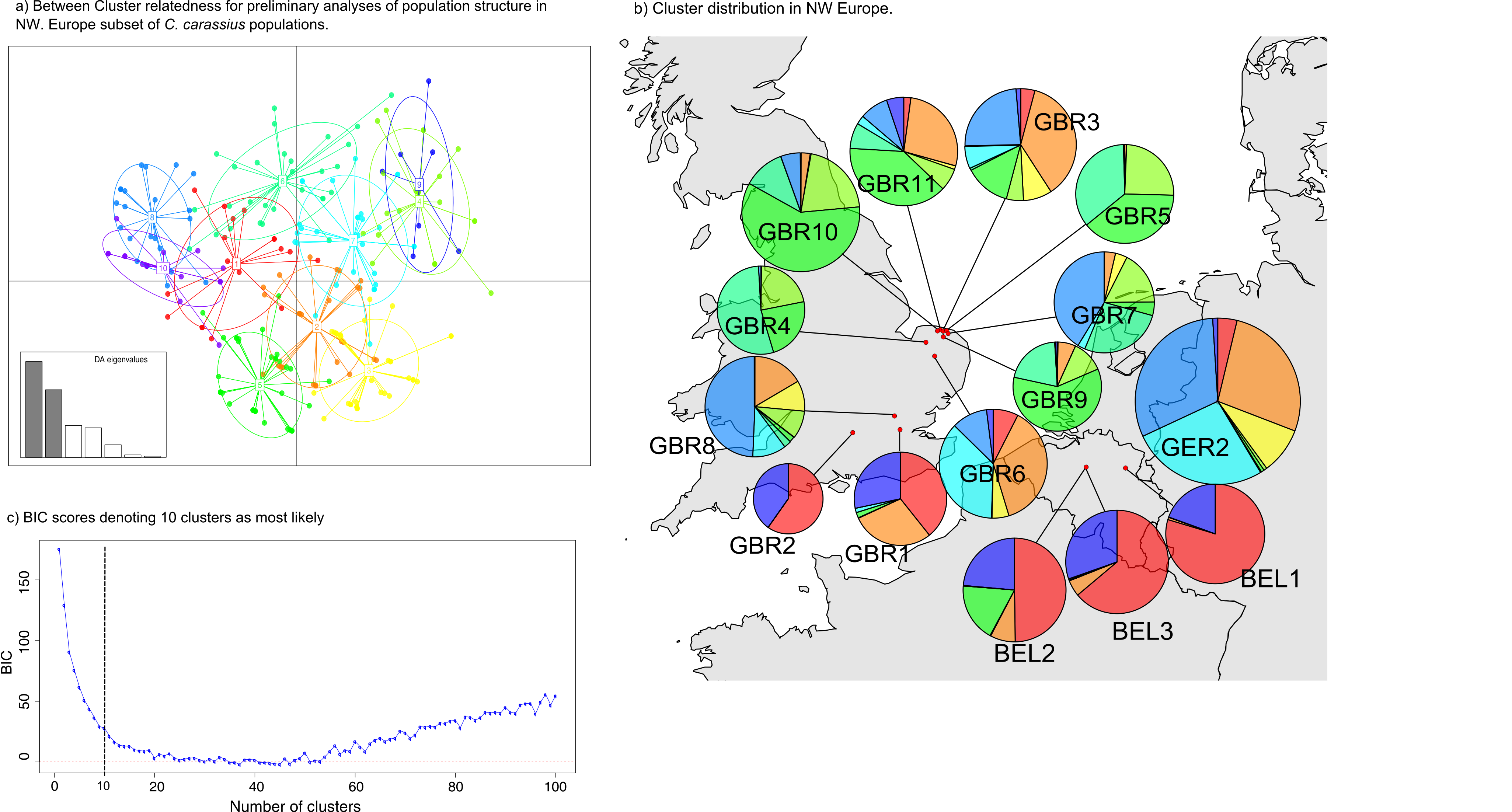


Figure S5. DAPC analysis of English, German and Belgian *C. carassius* populations: a) Shows relatedness between inferred clusters, b) shows geographic distribution of those clusters within populations and c) gives the BIC scores denoting ten clusters as the most likely (the number of clusters after which no significant change in BIC score is observed).
